# Use of point-of-sale data to track usage patterns of residential pesticides: methodology development

**DOI:** 10.1186/1476-069X-5-15

**Published:** 2006-05-25

**Authors:** Nyree Bekarian, Devon Payne-Sturges, Stuart Edmondson, Bill Chism, Tracey J Woodruff

**Affiliations:** 1 US Environmental Protection Agency, Public Health and Environmental Policy Team, National Center for Environmental Economics, 75 Hawthorne St, MC PPA-1 San Francisco, CA 94105, USA; 2 US Environmental Protection Agency, Office of Children's Health Protection, 1200 Pennsylvania Ave., NW Ariel Rios Bldg MC 1107A Washington, D.C. 20004, USA; 3 Kelly Registration Systems, Inc. 10115 Hwy 142 N. Covington, GA 30014, USA; 4 US Environmental Protection Agency, Office of Pesticide Programs, Biological and Economic Analysis Division, 1200 Pennsylvania Ave, NW MC 7503C, Washington, D.C. 20460, USA

## Abstract

**Background:**

Residential-use pesticides have been shown to be a major source of pesticide exposure to people in the United States. However, little is understood about the exposures to household pesticides and the resultant health effects. One reason that little is known about home-use pesticide exposure is the lack of comprehensive data on exposures to pesticides in the home. One method to help ascertain the amount of pesticides present in the home is use of point-of-sale data collected from marketing companies that track product sales to obtain the volume of pesticides sold for home-use. This provides a measure of volume of home-use pesticide.

**Methods:**

We have constructed a searchable database containing sales data for home-use permethrin-containing pesticides sold by retail stores in the United States from January 1997 through December 2002 in an attempt to develop a tracking method for pesticide. This pilot project was conducted to determine if point-of-sale data would be effective in helping track the purchase of home-use permethrin containing pesticides and if it would stand as a good model for tracking sales of other home-use pesticides.

**Results:**

There are several limitations associated with this tracking method, including the availability of sales data, market coverage, and geographic resolution. As a result, a fraction of sales data potentially available for reporting is represented in this database. However, the database is sensitive to the number and type of merchants reporting permethrin sales. Further, analysis of the sale of individual products included in the database indicates that year to year variability has a greater impact on reported permethrin sales than the amount sold by each type of merchant.

**Conclusion:**

We conclude that, while nothing could completely replace a detailed exposure assessment to estimate exposures to home-use pesticides, a point-of-sale database is a useful tool in tracking the purchase of these types of pesticides to 1) detect anomalous trends in regional and seasonal pesticide sales warranting further investigation into the potential causes of the trends; 2) determine the most commonly purchased application types; and 3) compare relative trends in sales between indoor and outdoor use products as well as compare trends in sales between different active ingredients.

## Introduction

The United States Environmental Protection Agency (EPA) estimates that production of pesticides tops 1.1 billion pounds annually, amounting to about $11 billion in sales [1]. In the United States about 900 pesticide active ingredients are registered for use with the EPA [2] and over 20,000 pesticide products are available for agricultural, commercial, and household uses [1]. An estimated 90% of all households in the country use pesticides, accounting for 74 million pounds for which consumers pay $1.2 billion per year [1]. Several studies have shown that residential-use pesticides are a major source of human exposures to pesticides [3,4], yet human exposures to residential-use pesticides are poorly understood.

Pyrethroid pesticides, synthetic versions of the naturally occurring pesticide pyrethrin which is derived from the chrysanthemum flower, are the most commonly used active ingredients in over-the-counter pesticide formulations [5]. Pyrethroids have largely replaced organophosphate pesticides (OPs) [6], which, until recently, were the most widely used active ingredients found in household pesticide formulations [7]. The EPA began restricting use of OPs in and around the home in 2000, citing risk of human toxicity upon exposures to the compounds, especially to children [8]. The EPA also initiated a phase-out of OPs as active ingredients in pesticides intended for outdoor home-use by 2004 [8].

Unlike OPs, pyrethroid pesticides have low acute toxicity in humans and have long been thought to be safe for human exposure [9]. However, although their acute toxicity to humans is low, studies have shown that pyrethroids can produce chronic health effects such as immunotoxicity, neurotoxicity, and endocrine disruption in humans and other mammals [10-13].

Permethrin is the most common pyrethroid derivative used in residential pesticide formulations [14]. First registered for use in 1977 [2], it is effective against head lice, fleas, ticks, mites, cockroaches, and other pests [15]. Permethrin tends to be relatively persistent and can remain active for several weeks after application [16]. It is rapidly degraded in mammalian systems and, although it is lipophilic, permethrin does not bioaccumulate in the human body [5]. Exposure to permethrin in the home can occur through inhalation, ingestion, and dermal absorption.

### Tracking home-use pesticides

While use and exposure to pesticides applied in agricultural settings have been studied more thoroughly, less is known about the uses, exposures, or human health effects of pesticides used in or around the home [17], especially with respect to products purchased over-the-counter (pesticide products that can be purchased and applied without the need for professional licensure) by consumers. Because of the variability in uses and application practices of over-the-counter pesticides used in the home there is a considerable amount of difficulty in estimating the amount of pesticide used or the nature of exposures that occur and, therefore, in estimating the potential risk to human health.

There have been several attempts at tracking pesticide use in the United States, each with varying degrees of success. At the state-level efforts include pesticide tracking systems in California, New York, Massachusetts, New Hampshire, and Oregon. These systems require Pesticide Use Reporting (PUR) and include agricultural as well as non-agricultural applications (including commercial and institutional applications, and professionally applied residential pesticides) [18]. Of those, Oregon is the only state that requires tracking of household pesticides available "over the counter", which is done through the use of point-of-sale reporting; though Oregon's program is currently on hold due to the state's fiscal crisis [18,19]. New York has the most comprehensive tracking system in place, allowing for the characterization of urban-use pesticides by commercial applications. This system requires applicators to supply information on type and quantity of pesticide used for any professionally applied pesticides as well as the address where the pesticides were applied [18]. Public access to this database is restricted.

National level tracking systems include survey and measurement-based studies. The Centers for Disease Control's (CDC) National Health and Nutrition Examination Survey (NHANES) survey, a voluntary questionnaire-based survey, has added questions on residential pesticide use in their 1999–2000 survey [20], but the survey lacks questions about specific active ingredients, pesticide formulations, or types. EPA's Non-Occupational Pesticide Exposure Study (NOPES) was a pilot study, aimed at developing monitoring instrumentation, laboratory methods, and survey questionnaires to conduct a Total Exposure Assessment Methodology (TEAM) for pesticide exposures. The end goal of the study was to develop a framework for estimating people's use of and exposures to pesticides and to take accurate measurements of pesticides in variety of media, such as air, water, and food as a reference point for estimates made from questionnaires. Data were collected from volunteers residing in two cities in three phases and results were reported on in a report published in 1990 [21]. NOPES did not attempt to determine the source of pesticide exposures; however it did make headway in assessing the most important mediums for monitoring pesticide exposure, helped to refine questionnaires to better estimate pesticide use in the home, and yielded quantitative estimates of pesticide concentrations in the air, and qualitatively estimated water, food, and dermal exposures.

The EPA has also attempted to understand residential exposures to multiple chemicals, including pesticides, through community-based and regional scale studies such as the National Human Exposure Assessment Survey (NHEXAS) [22]. This voluntary study, begun in 1995 [23], investigated residential exposures to humans to multiple chemicals, including residential-use pesticides, mainly chlorpyrifos and diazinon (another OP). This was done by taking measurements of these chemicals in the home environment (sampled medium included air, house dust, soil, drinking water, and food) [24] and collecting data through the use of questionnaires in an attempt to identify predictors of exposure at a population level, establish new modeling techniques, and understand what chemicals or combination of chemicals pose the greatest human health risks. The studies conducted within NHEXAS had a specific set of studies concerning exposures to children, the Minnesota Children's Pesticide Exposure Study (MNCPES) [25]. Major findings from these studies showed that common pesticides found in homes included chlorpyrifos, permethrin, and pyrethrin [26,27]. Of the houses that were tested, chlorpyrifos was found in 92.5% of the indoor air samples. It was also found in carpet dust, soil, and food samples [27]. Personal monitoring was done in at least one study conducted by Gordon et al and included dermal wipes (to test skin exposure) and personal air sampling. The study found chlorpyrifos more readily on skin than in personal air samples [24].

Before EPA takes regulatory actions on pesticides, studies are conducted on human health, environmental, economic, and social impacts as required by the Federal Insecticide, Fungicide, and Rodenticide Act (FIFRA) 92–515, as amended [28]. The EPA has evaluated and published information on pesticide sales for many years [1]. The last home and garden pesticide user survey that the EPA conducted was in 1992 and currently it is estimated that it would cost over a million dollars to repeat the survey [29]. However, to date, there is no comprehensive source of information available to the EPA on types and amounts of pesticides purchased by homeowners and renters in the United States. In this article we evaluate a national point-of-sale database for permethrin, a pyrethroid pesticide widely used in home pest management, as a novel approach to track over-the-counter sources that contribute to residential pesticide exposures. The data provided by this study can give a snapshot of pesticide usage and allows us to compare potential relative usage of pesticides between regions of the country. We hope to use this point-of-sale database to 1) help detect anomalous trend in regional and seasonal pesticide sales that may warrant further investigation as to the potential causes of the trend; 2) determine the most commonly purchased application types; and 3) examine relative trends in sales between indoor and outdoor use products as well as sales trends between different active ingredients.

## Materials and methods

Market research companies collect and maintain sales data for thousands of consumer items, including pesticide products for manufacturers and retailers. This information is tracked through the use of Universal Product Codes (UPCs), which serve as unique identifiers for each product manufactured. However, these marketing data often lack more detailed information on products like pesticides, such as formulation and percent active ingredient. In order to obtain information on amount of pesticide active ingredient sold to residential users for the purpose of this study we needed to link sales data for permethrin based products from one market research company with another data source that could provide information on active ingredient, percent active ingredient, pesticide type, formulation, and EPA registration number for each product sold. We then were able to match products to their assigned UPC codes then link the two data sources together in a customized dataset that contained all the necessary information for our investigation.

### Two data sources

#### Vista Information Systems

**Figure 1:**
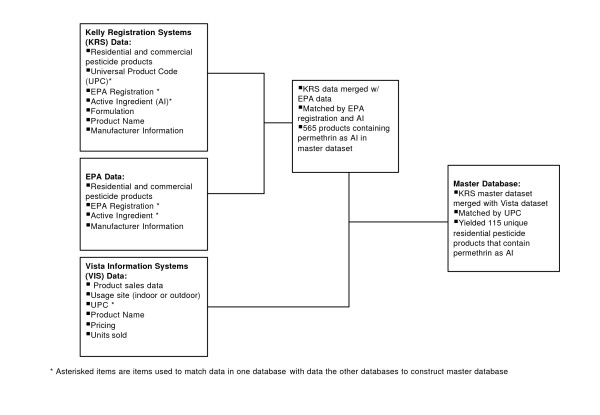
Flowchart illustrating the creation of a database for tracking volume of residential permethrin products sold through Hardware/Independent Retailers and Chain Homecenters.

Vista Information Systems (VISTA) provided point-of-sale data for all home and garden products containing permethrin and sold by hardware and home improvement retailers in the Unites States during the time period from January 1997 through December 2002. Vista's data, which covers non-commercial products intended for residential use, came from individual retail companies and stores who voluntarily supply information such as the container size, product type, and formulation (aerosol, fogger, powder) for each UPC. Since reporting to VISTA is voluntary, only a portion of all products sold in retail stores are represented by their data. The data that was provided by Vista included whether the product was intended for indoor or outdoor use, unit and pricing information (number of units sold, dollar values sold, and average unit price sold) categorized by region (National, Northeast, South, Midwest and West), and sales channel (a term used to define groups of retailers that provide similar types of products and operate on similar scales with respect to each other), which include Mass Merchants (major department-store chains), Chain Home Centers (major retail chains focused on home improvement products), Hardware Stores/Independents (smaller, independently owned franchises and Mom & Pop stores focused on home improvement products). Figure 1 illustrates the process by which the individual data sets were merged to create a master database.

Data reported to Vista from 1997 to 2002 initially included sales data from all companies that traditionally reported to the company; however a large retail company reporting in the mass merchant channel ceased reporting to Vista in July of 2001 and reports we obtained from Vista omitted data from this particular mass merchant company beginning in January, 2000.

#### Description of sales channels

Point-of-sale data maintained by Vista can be broken down into regional categories, representing the 48 contiguous states and the District of Columbia: Northeast, South, Midwest, and West Table 1.

**Table 1:** States represented by each sales region

**Sales Region**	**State**
Northeast	ME, VT, NH, MA, RI, CT, NY, NJ, PA
South	DC, DE, MD, WV, VA, NC, SC, KY, TN, GA, FL, AL, MS, TX, OK, LA, AR
Midwest	ND, SD, NE, KS, MN, IA, MO, MI, WI, IL, IN, OH
West	WA, OR, ID, MT, WY, CA, NV, UT, CO, AZ, NM

The national category includes a summation of the three sales channels across all four regions of the contiguous 48 states. While all three sales channels report to the national category, the regional data only reflects data obtained for the hardware/independent and chain home center channels. This difference is due to reporting trends and practices within the retail industry. Because of the major changes in reporting trends that occurred in the mass merchant channel during this study period, as stated above, all data presented in this report reflect the omission of the mass merchant channel from the analysis of the national sales channel.

#### Kelly Registration Systems

Kelly Registration Systems (KRS) currently maintains a database of all registered pesticide products from 33 states, which is updated electronically by the states on a regular basis. The data KRS receives contains product names, EPA registration numbers, manufacturer information, percent active ingredient, EPA registration status (approved, cancelled), and an expiration date for licensure. This data remains unchanged as it is published for consumer use on a website maintained by KRS; however, it is supplemented with data from the EPA such as: active ingredients (AI), percentage of AI for each EPA registration number; a list of pests controlled by each EPA registration number, a list of sites/crops to which each EPA registration number is approved for use, a list of pesticide types for each EPA registration number, and a single formulation for each EPA registration number. Although the information is routinely updated, it is KRS policy to not alter data that are provided by EPA or individual states. Manufacturers who use the database are able to match their products to a common product equivalent by referencing another database within the system. This allows identical products to be matched even if they have different names, which can vary depending on state or region of the product's origin. All data conversions are done electronically and quality assurance queries are performed to ensure that the electronic conversion was completed successfully, however, no manual data-entry or data editing is performed.

### Identifying products containing permethrin

Products containing permethrin for this study were identified in a two-step process. A dataset provided by Vista consisted of 45 products that the marketing firm believed to contain permethrin as an active ingredient. The dataset was found to be extensive but not complete for all products containing permethrin. KRS then obtained a master list of all UPCs from Vista with associated product names and cross-referenced the list with a large database of UPCs maintained by KRS in order to find the 565 actively registered products containing Permethrin as an AI. KRS manually 'updated' its UPC database with the information from Vista and screened and edited the data for duplicates and mismatched product names. KRS was then able to extract a list of UPCs that contain Permethrin, which were supplied to Vista so they could extract sales information for those relevant UPCs. This resulted in a total of 115 unique UPCs coding for products that contain permethrin as an AI.

### Merging datasets

Several queries were developed to join the data and to create 'temporary' tables. These steps ensured that, as the EPA data is updated on a monthly basis, the web-based database would be overwritten with up-to-date EPA data, allowing it to stay current with minimal effort

KRS then merged its list with Vista's by matching UPC numbers, which resulted in a dataset containing quantity of units sold by month, container size, region, sales channel, and percent active ingredient as well as other pertinent information for the time period from Jan '97-Dec '02 Table 2. This became the master database from which we were then able to extract the desired information.

**Table 2:** Definitions of terms used in product information descriptions

**Product Information**	**Definition**
UPC number	Universal Product Code – a string of 12 numbers that acts as a unique identifier code for each product; represented by a bar code on most retail products
Item description	Short description of product type and brand
Manufacturer	Company that manufactures and markets product under brand name
Brand	Product name under which it is sold by retailers
Product Type	Describes type of pesticide, in this case insecticide
Usage Site	Describes whether product is intended for indoor or outdoor use
Pest Type	What type of insect product is intended to be used on
Application Type	Method of deliver, whether product is an aerosol, fogger, powder, etc
Container Type	Type of container insecticide is sold in: trigger sprayer, aerosol can, trap, etc.
Container Size	Weight of container or size of package
Sales Channel	Identifies whether product was sold at a Hardware Store, Home Center, or Mass Merchant
Region	Identifies if product was sold in the Northeast, South, Midwest, or West
Percent Active Ingredient	Describes percentage of active ingredient (permethrin) in product
Date of Sale	Describes Month and Year of sale of product
Price	Amount Sold in Dollars

The dataset was prepared for descriptive analysis by ensuring all units of measurement and price were uniform or convertible to uniform units. Most of the data conformed to these parameters; however, there were a few exceptions. Some UPCs were associated with container sizes that were in units like UNIDENT, N/A, CT, or PK, as opposed to ounces or pounds, were not convertible into units of measure. Because units for these products were non-convertible, it was necessary to drop these products from the dataset. Roughly 17% of the data was dropped. These data were evenly distributed across the regions and sales channels; therefore we did not expect this to have any impact on our analysis.

The data were then sorted by region (National, Northeast, South, Midwest, and West) and exported into MS-Excel for analysis.

## Results

### Pounds sold

**Figure 2:**
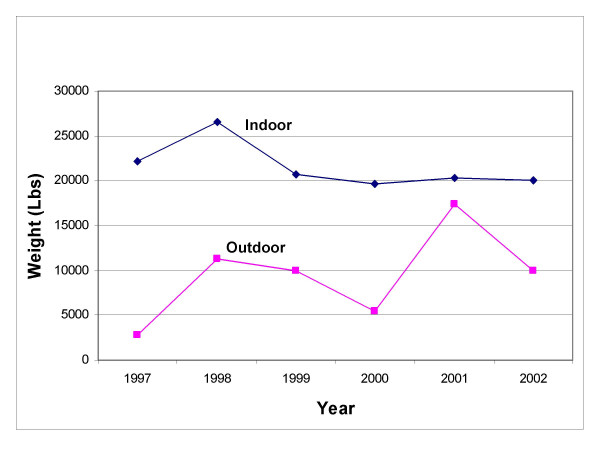
**Total Pounds of permethrin sold nationally as estimated by the database. **Total pounds of permethrin sold annually in indoor-use and outdoor-use products from 1997–2002.

With the exception of a small increase in pounds of active ingredient sold in 1998, the national sales trend for pounds of indoor-use permethrin products sold was relatively level for the entire study period. The trend for outdoor-use permethrin pesticide was more dynamic. Nationally, there was a three-fold overall increase in total pounds AI for outdoor-use permethrin-based pesticides sold from 1997–2002 (Figure 2); increasing from 2700 lbs sold in 1997 to 10,000 lbs in 2002.

**Figure 3:**
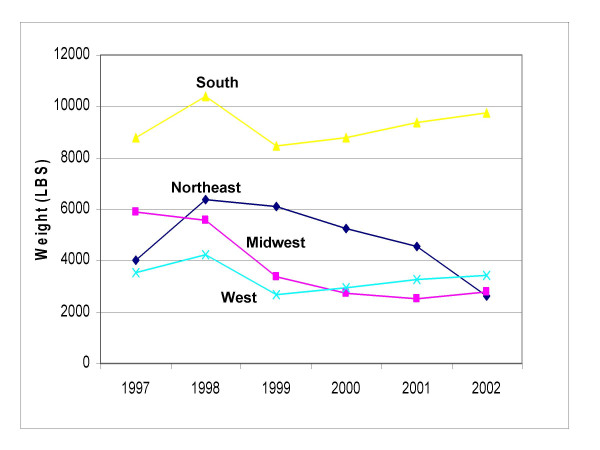
**Pounds of permethrin sold for indoor-use by region as estimated by the database**. Total pounds of permethrin sold in indoor-use products from 1997–2002, as determined by percent of permethrin active ingredient in each product and total weight of each product.

There was an overall decrease in pounds AI for indoor-use products sold regionally from 1997–2002 (Figure 3). The Midwest experienced the greatest decrease in sales from 1997 to 2002. Indoor-use pesticide sales in the South were an exception to the regional sales trends in pounds (lbs) AI sold during the study period, increasing by about 11%.

Sales in pounds AI increased in all regions for outdoor-use pesticides for this study period. The largest increase was for the South. In 2001 sale of permethrin-based outdoor-use products in the South spiked from about 2000 lbs to almost 13,000 lbs. These numbers contributed to 74% of total nationwide sales for outdoor products for that year. In 2002, sales in the South contributed to just over half of total nationwide sales for that year.

### Units sold

**Figure 4:**
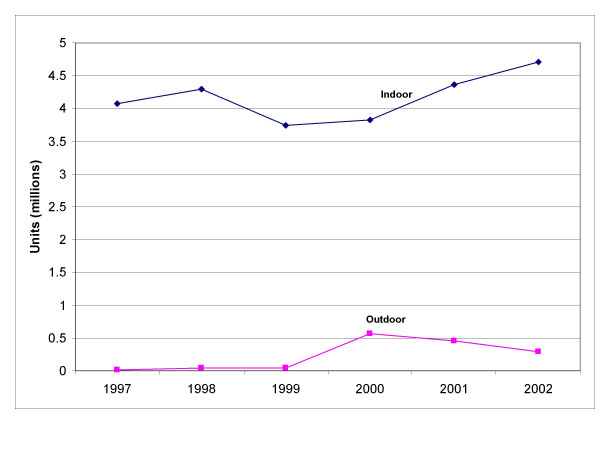
**Units of permethrin-containing products sold nationally as estimated by the database.** Total number of units of permethrin containing products sold per year from 1997–2002.

'Sale in units' is another method of assessing total sales of pesticide products. A unit is a metric by which a product is packaged. Unit sales on the National scale increased for both indoor-use and outdoor-use products from 1997–2002 (Figure 4). Unit sales of indoor-use permethrin products increased steadily from about 4 million units to about 4.8 million units during the study period, an increase of about 15%. Sales of outdoor-use permethrin products had a higher relative growth rate than indoor-use products during the study period, from 16,800 units sold in 1997 to about 870,000 units sold in 2002.

Overall sales in units for indoor-use permethrin products increased slightly in all regions except for the Midwest. Sales in the South increased the most from 1997 to 2002, starting at 1.5 million units and ending at 2 million units of pesticide sold per year. The Northeast and West experienced modest increases in sales.

**Figure 5:**
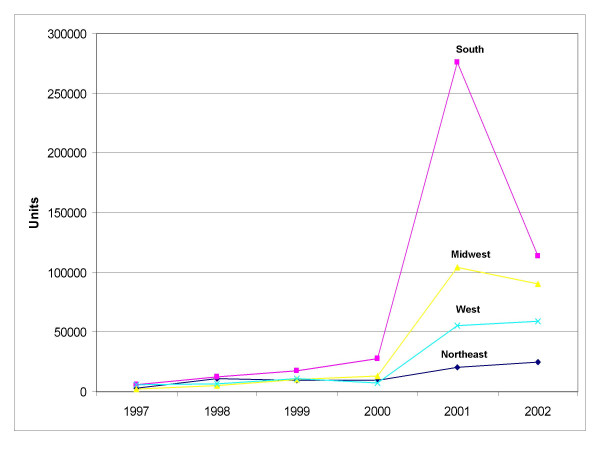
**Units of permethrin-containing products sold regionally for outdoor use as estimated by the database.** Total number of units of outdoor use permethrin containing products sold per year from 1997–2002 for each of the four regions of the database.

Overall regional sales in units for outdoor-use permethrin-based pesticide products increased for all regions during the study period (Figure 5). In 2001 sales in each region increased markedly, with the most dramatic spikes in unit sales occurring in the South, which increased from 28,000 units sold in 2000 to 275,000 units sold in 2001. The Midwest also had increases in unit sales during the study period. From 2001 to 2002 sales in the South decreased by 59%, but remained level for the other regions for the same time period.

### Indoor vs. outdoor

More pounds of AI and more units of indoor-use permethrin-based pesticide products were purchased than outdoor-use products. Total nationwide sales in dollars of indoor-use products were $26.2 million, whereas total sales of outdoor-use products were $3 million (data not shown). Similar trends existed for sales in pounds and sales in units where sales of indoor-use products were much greater than sales of outdoor-use products for both units of measure.

Analysis of the data on a regional scale also showed that indoor-use permethrin products were more popular than outdoor-use permethrin products; sales for indoor-use products were consistently higher than sales for outdoor-use products in all four regions from 1997–2002.

### Seasonal trends

Seasonal sales trends for the four regions of the study were similar to each other. Sales of permethrin-based products peaked during the summer months with slight temporal variability by region, which may be attributable to the climatic differences in each region. The combined result for the overall national seasonal trend showed sales of permethrin-based products peaking in mid-July to August and dropping to a low during the winter months.

### Application types

**Figure 6:**
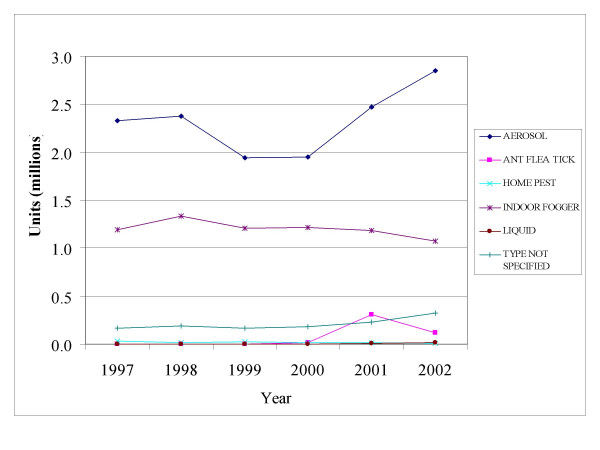
**Units of pesticide product sold by type as estimated by the database.** Total unit sales of indoor-use permethrin products from 1997–2002. Products are broken down by application type.

Of the different application types examined the two most commonly purchased for indoor-use permethrin based pesticides were aerosol sprays and indoor foggers (Figure 6). National sales of aerosol products by unit were higher than sales for indoor foggers for the entire period from 1997–2002.

Pound for pound, sales of aerosols and indoor foggers were comparable from 1997 through 2000. In 2001, sales in pounds of aerosols rose above sales of foggers, which remained level throughout the study period.

National sales in dollars of indoor fogger were greater than sales for aerosols from 1997 through 2002. In 2001, sales in dollars of aerosol pesticides containing permethrin surpassed sales of indoor foggers containing permethrin. By 2002, annual sales of aerosols topped $11 million.

### Market trends

The sale of permethrin-based pesticide products experienced a slight increase, overall, for both indoor-use and outdoor-use products during the study period. The national sales average increased from $3.2 million to about $4.1 million over the 6-year period. Sales increases for indoor-use and outdoor-use products were driven by large increases in sales in the South, especially when annual sales in this region increased from $780,000 in 2000 to about $3.5 million in 2001.

## Discussion

A market-based approach to tracking sales of residential-use pesticides is a unique way to monitor patterns and trends in non-commercial, home use of these pesticides. This paper illustrates the type of information that can be obtained from point-of-sale data. The data provided by this study gives a snapshot of pesticide usage and allows us to compare potential relative usage of pesticides between regions of the country. In this study, there are three major areas of comparison that were examined: purchase of indoor use vs. outdoor use pesticide products, regional differences in pesticide purchases, and most commonly purchased application types.

A study comparing indoor vs. outdoor sales of permethrin-based products gives a basis on which to focus a more detailed study, potential exposure assessment, or look more closely at the potential toxicological risks associated with use of products that are purchased more frequently. Similarly, looking at regional differences in buying patterns can tell us if one region of the country may be getting a higher exposure to a pesticide compared to the rest of the country. We can also potentially use this information to compare trends in pesticide purchasing with events involving large-scale pest invasions or outbreaks of diseases that are spread by biting insects and other pests. The formulation piece of the study is important because it helps to identify relevant routes of exposure (inhalation, dermal, etc).

Permethrin's chemical properties and toxicokenetic mechanisms in mammals make it an appropriate alternative to OPs. It's chemical properties are why permethrin is often preferred as an active ingredient in pesticide products intended for indoor use over other ingredients, such as carbamates. This is reflected in the sales trends for over-the-counter permethrin products from 1997–2002, which increased over time. Also, sales in units, pounds, and dollars for indoor-use permethrin-based pesticides were much greater than for outdoor-use products during this time-period.

It is not surprising that different regions in the United States have different sales of pesticide products. The South, being a temperate and humid climate where indoor infestations may be a problem, may have a greater need for indoor pesticides than a colder climate area such as the Northeast or a drier climate area such as the West. This may explain why sales for indoor-use pesticides were higher in the South compared to the Northeast, Midwest, and West. While this trend is likely dictated by a region's need for pesticide use, there are some anomalies in the data that are not easily explained by regional weather, such as the large spike in sales for outdoor-use permethrin product that was seen in the South in 2001. Because the sales data that we obtained gave us a snapshot in time were are able to identify this anomaly, look back to events occurring in 2001, and investigate what may have been happening in the South at that time. For example, we can use this type of database to look at use of pesticides as a response to West Nile Virus (WNV). According to the CDC the first cases of WNV were reported to have occurred in humans in the South in 2000 [30]. By 2001, most of the southern states reported human cases of WNV. The increased purchase of outdoor-use residential pesticides in the South in 2001 could potentially be explained by people reacting to the arrival and spread of WNV in the region.

Additional study is required to prove that the spike in sales for outdoor-use permethrin-based pesticides in 2001 in the South is attributable to the spread of WNV. However, the WNV story exemplifies how point-of-sale data can be used to pin point interesting trends in pesticide usage that may warrant closer attention.

Another trend demonstrated by the point-of-sale database was the overall increase in national sales in units of both indoor and outdoor-use permethrin-based pesticides. This increase be explained by the restrictions put on chlorpyrifos and other OPs by the EPA. In 2000, the US EPA restricted the use of chlorpyrifos, the most widely used homeowner pesticide active ingredient, from products intended for indoor household-use [31]. The Agency also announced a phase-out and eventual cancellation of diazinon, another OP, for outdoor, residential use [32], adding to the growing list of OPs that had been cancelled for such uses. As of 2000, only commercial and agricultural applications of chlorpyrifos would be allowed, and all sales and applications of these chemicals would have to be recorded and tracked through the EPA. This move came as a result of an extensive review of chlorpyrifos, which included studying its toxicity to humans [33]. Pyrethroids and, to a lesser extent, carbamates have replaced OP pesticides [6], thereby potentially explaining the upward trend in permethrin sales in units seen from 1997–2002.

It is interesting to note the differences in sales trends using weight in pounds of permethrin sold compared to the units of permethrin products sold. While, in theory, the trends for the two measures should be similar, the sales trends for units sold increased overall from 1997 to 2002 and decreased for weight of active ingredient sold. These two measures may be different because of altered formulations, such as formulations where two pyrethroids are mixed and a lower rate of permethrin is used or where there has been the addition of a synergist. Formula alterations may result in a need for less active ingredient for a product to be effective. The ensuing outcome would be less pesticide by weight sold per year. At the same time, an increased demand for a product would raise sales, resulting in an increase in the number of units sold per year. This is an important finding that illustrates the fact that all questions involving pesticide sales trends may not be addressed intuitively. Different queries must be explored in order to accurately interpret the sales trends and other data.

There are several issues involved with utilizing point-of-sale marketing data to track pesticide usage patterns. The greatest challenge comes from availability of sales data. Not all merchants are willing to report sales information to marketing companies, which can result in underestimates of pesticide use. One such company was reporting its sales data to marketing companies and then ceased reporting in July 2001. Vista omitted this company from its market reports from January 2000 on because the market information for that year was incomplete. When this large retail chain in Vista's mass merchant channel discontinued the reporting of their sales data to Vista in 2001 it had a profound effect on total, national sales figures. The difference in reported sales in pounds of permethrin dropped from just over 45,000 lbs of active ingredient sold in 1999 to 3,512 lbs sold in 2000; a 14-fold difference. If reported sales of permethrin in pounds continued on the established trend from 1997–1999 in Vista's mass merchant channel, a roughly linear trend, then projected sales in pounds in 2002 would have been about 58,000 lbs, vs. the 4,051 lbs reported by Vista. As a result, combined projected national sales for that year from Vista would have been about 88,000 lbs, which is roughly 3 times as much as observed in Vista's home center and hardware channels combined.

Since the majority of total sales in the mass merchant channel are attributable to this company, dropping this company's data in 2000 led to a 14-fold decrease in reported total sales for that year, complicating the analysis and leading us to drop the mass merchant channel completely from our national permethrin sales analysis. If the goal of using point-of-sale data were to extrapolate actual pesticide exposures in people using residential pesticides, the estimated exposures would fall extremely short of the actual exposures without availability of the mass merchant sales channel. However, the purpose of this pilot project was for pesticide use tracking and comparing relative, not absolute, usage by region and season. Therefore, we were able to continue the analysis without data from the mass merchant channel; the assumption being that sales trends within the mass merchant channel would mirror trends in the chain home centers and independent hardware channels. We checked this assumption by comparing trends between the channels for the years where there was complete information. Patterns for regional and seasonal sales, trends in sales of popular formulations, and ratios of indoor to outdoor sales were similar across each of the sales channels for the years with complete information. Therefore, despite the exclusion of the mass merchant channel, we were able to analyze this pilot sales database for potential trends in sales for a small share of the market (e.g. the home center and hardware store channels reporting to Vista). A next step following our pilot study would be pesticide use surveys to confirm the trends observed in the point-of-sale data from Vista, which represents a small fraction of the market. This would help us determine if the point-of-sale data are adequate indicators for residential pesticide purchases and use.

Another issue with tracking using point-of-sale data is market coverage. With the exception of the mass merchant channel, much of the remaining home consumer market is covered by the Vista survey. Components of the market that were excluded from the Vista survey were lumberyards, lawn and garden stores (i.e. nurseries), club stores, and grocery stores, which either do not report their sales data to any marketing agencies or report their data to agencies other than Vista. Veterinary clinics were also not included in market data. This area may be important because permethrin is used in many flea and tick preparations for dogs and cats.

Resolution is another problem with the market-based approach. Most marketing companies do not report their sales data at a geographic level any finer than a wide regional level, such as Northeastern or Southern states, leaving no ability to examine sales by county. This greatly limits our ability to find areas of higher or lower pesticide sales, which creates broad generalizations of regional usages and, therefore, exposures

Finally, data tracked by marketing companies are not part of public databases. Access to these databases is limited, at best, as companies are often reluctant to make information public by selling their data. While not as big of a problem for the public at large, government organizations like EPA become severely limited in the type and quality of information that is available.

Permethrin is only one out of hundreds of pesticide active ingredients that are used in residential pesticide formulations. It was chosen for this study as a benchmark pesticide because of its widespread use. In order for a pesticide tracking system to be completely successful, it will be necessary to obtain point-of-sale data for all pesticides formulated and sold for home-use. With this in mind, it is important to also note that pesticides purchased over-the-counter are not the only source of pesticide exposure in the home. Many common household items come pre-treated with pesticides, usually permethrin or other pyrethroids. Rugs, mattresses, curtains, and other similar items may be treated in factories to proof them from moths and other pests [34]. Other sources of pesticide exposure include dietary exposures from food, as well as drift from agricultural applications and pesticides that are tracked into homes by individuals exposed in occupational settings. It is not clear how significant pesticide exposures related to these sources are. What this point illustrates, though, is that pesticide use is more widespread than many people may think. While a point-of-sale tracking system could not fully represent all aspects of the home-use pesticide market, it could be a useful tool in the overall effort to understand these exposures.

## Conclusion

Residential environment and consumer products, such as pesticides, have been identified as significant contributors to human exposures and health risks. While all agricultural and commercial pesticides used in the United States are regulated by the EPA, it is considerably more difficult to control the use of over-the-counter pesticides once they are purchased by the consumer. Products intended for home use must be registered with EPA but use reporting, i.e. how much of a product is used, when, and by whom, is not readily available. As a result, it is difficult to assess the amount of exposure to pesticides that people experience in their homes; therefore, exposures are very hard to control. Because performing exposure assessments for every household pesticide active ingredient available in the United States would be logistically difficult and expensive, having the ability track use of these pesticides by employing pesticide sales data could be a practical alternative. Despite limitations associated with pesticide tracking through point-of-sale data, such as availability of sales data, market coverage, and geographic resolution, this type of database is still sensitive to relative trends in pesticide sales. While not without its problems, creating a point-of-sale database would provide a helpful snapshot of usage patterns from region to region, allowing us to locate areas of the country that may warrant more detailed investigations and further helping us understand human exposures to home-use pesticides.

## Abbreviations

EPA Environmental Protection Agency

OP Organophosphate Pesticides

PUR Pesticide Use Reporting

CDC Centers for Disease Control

NHANES National Health and Nutrition Examination Survey

NOPES National Occupational Pesticide Exposure Study

TEAM Total Exposure Assessment Methodology

NHEXAS National Human Exposure Assessment Survey

MNCPES Minnesota Children's Pesticide Exposure Study

FIFRA Federal Insecticide, Fungicide, and Rodenticide Act

UPC Universal Product Code

VISTA Vista Information Systems

KRS Kelly Registration Systems

WNV West Nile Virus

## Competing interests

The author(s) declare that they have no competing interests.

## Authors' contributions

NB analyzed and interpreted database, drafted article

DPS conceptualized study and design, involved in drafting and revising article, final approval of published article

SE developed design methods and constructed database

BC involved in revising article critically for important intellectual content

TJW contributed to development of study design and revising article
